# Processing load, and not stimulus evidence, determines the duration of unconscious visual feature integration

**DOI:** 10.1038/s44271-023-00011-2

**Published:** 2023-08-08

**Authors:** Lukas Vogelsang, Leila Drissi-Daoudi, Michael H. Herzog

**Affiliations:** Laboratory of Psychophysics, École Polytechnique Fédérale de Lausanne (EPFL), Lausanne, Switzerland

**Keywords:** Human behaviour, Visual system

## Abstract

Integration across space and time is essential for the analysis of motion, low contrast, and many more stimuli. A crucial question is what determines the duration of integration. Based on classical models of decision-making, one might expect that integration terminates as soon as sufficient evidence about a stimulus is accumulated and a threshold is crossed. However, there is very little research on this question as most experimental paradigms cannot monitor processing following stimulus presentation. In particular, it is difficult to determine when processing terminates. Here, using the sequential metacontrast paradigm (SQM), in which information is mandatorily integrated along motion trajectories, we show that the processing load determines the extent of integration but that evidence accumulation does not. Further, the extent of integration is determined by absolute time instead of the number of elements presented. These results have important implications for understanding the time course and mechanisms of temporal integration.

## Abstract (web summary)

When information is spatiotemporally integrated along motion trajectories, visual integration lasts longer with increased processing load. The number of visual elements does not appear to play a role for visual integration.

## Introduction

Long-lasting visual feature integration is crucial for detecting and integrating motion signals as well as for solving many of the ill-posed problems of vision. However, very little research has attempted to closely characterize these processes as most experimental paradigms do not allow to control the time course of temporal integration. Once a stimulus is presented, the experimenter has little control over the subsequent neural processes. In particular, it is difficult to determine when stimulus processing terminates. The sequential metacontrast paradigm (SQM), to the contrary, has turned out to be a versatile tool for examining the dynamics underlying temporal integration.

**Fig. 1:**
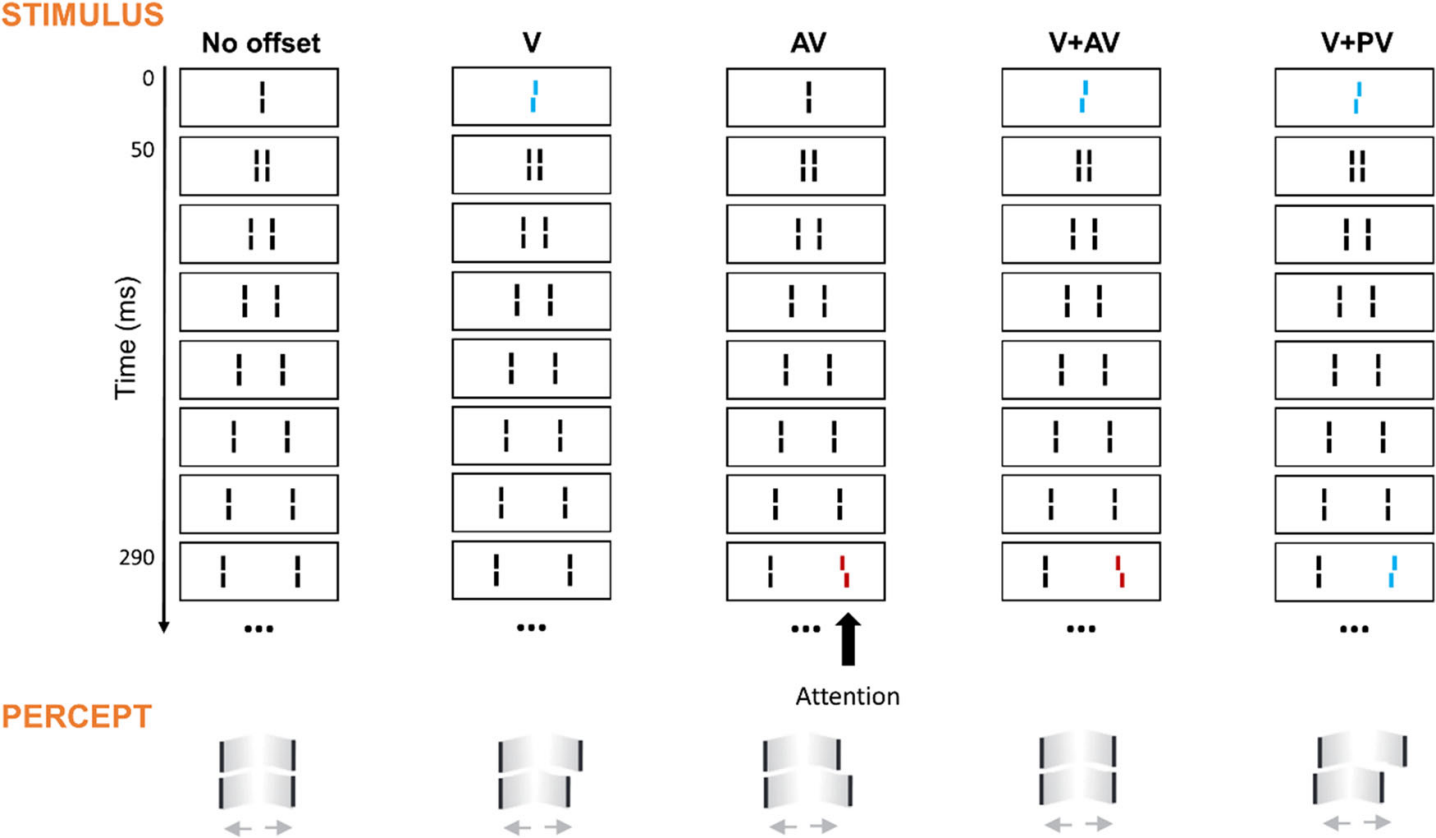
The sequential metacontrast paradigm (SQM). A central line is presented, followed by pairs of flanking lines, eliciting the percept of two diverging motion streams. Participants are instructed to attend to one of the motion streams (here, the right one) and report the perceived direction of the offset. If one of the lines in the stream is offset (e.g., the central line or a later flanking line), the entire stream is perceived as offset, even though the other lines are, in fact, straight. If both the central and a flanking line are offset, the two offsets integrate, if separated by less than about 300 ms, depending on the observer^2^: if the two offsets are in opposite directions, they cancel each other out before reaching consciousness; if they are in the same direction, the offset direction is perceived at increased performance levels. Note that the colors are only used for illustration. In the real experiment, all lines were white on a black background.

In the SQM^1^, participants are presented with a central line followed by pairs of flanking lines, eliciting the percept of two motion streams diverging from the center (see Fig. 1 for illustration). Due to metacontrast masking, the central line is rendered invisible. If the line is offset (i.e., the lower segment is shifted either towards the right or left, relative to the upper segment; here referred to as ‘vernier’ offset or ‘V’), participants perceive the subsequent flanking lines as offset, even though they are, in fact, straight. If, in addition to the central line, one of the flanking lines is offset, the two offsets integrate. If both offsets are in the same direction ('provernier’ or ‘PV’), the offset is perceived at even higher performance levels. If, instead, they are in opposite directions (‘antivernier’ or ‘AV’), the offsets cancel each other out before reaching consciousness, even if vernier and anti-vernier are separated by up to 450 ms^2^. Observers are not able to report or perceive either vernier offset separately. Hence, integration in this paradigm is mandatory.

**Fig. 2:**
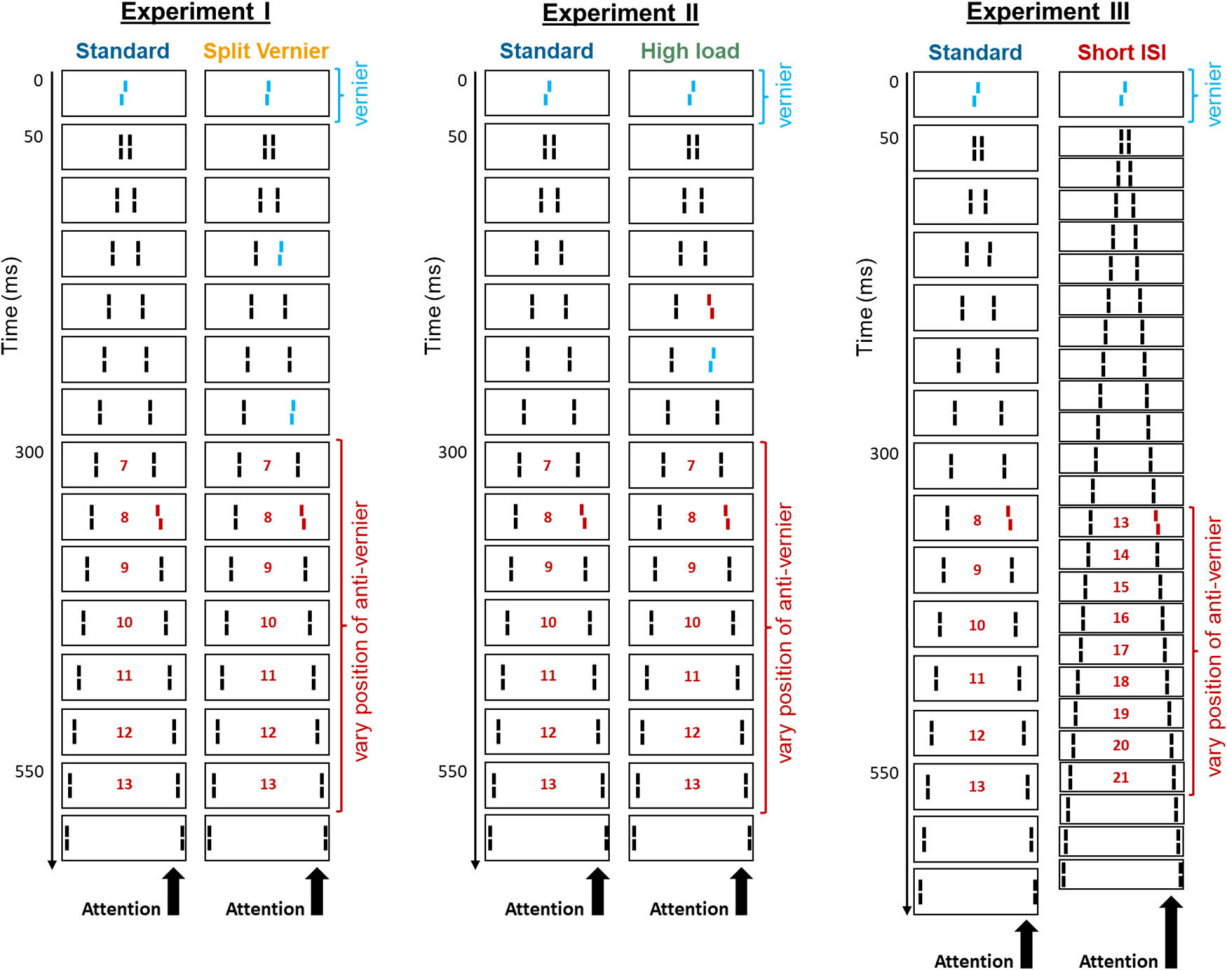
Illustration of different experiments. In all conditions, a central vernier was presented at the initial frame (frame 0). In the split-vernier condition of experiment 1, this initial vernier was smaller as additional small verniers were presented in frames 3 and 6. An additional anti-vernier was presented at either one of positions 7 through 13 in experiments 1 and 2, as well as at either one of positions 8 through 13 in the standard condition and at either one of positions 13 through 21 in the short-ISI condition in experiment 3. This systematic sampling was carried out to measure at which specific time points mandatory integration terminates.

Here, we asked what terminates stimulus processing and temporal integration. To this end, we conducted three experiments with the SQM, as illustrated in Fig. 2, and compared the resulting integration windows across different experimental conditions.

## Methods

### Participants

Naïve participants were recruited from the École Polytechnique Fédérale de Lausanne and the University of Lausanne in Switzerland. A total of 11 participants successfully completed experiment 1 (3 females; age range: 19–24 years), 11 (8 new, 3 old) participants successfully completed experiment 2 (2 females; age range: 19–26 years), and 15 (14 new, 1 old) participants successfully completed experiment 3 (5 females; age range: 18-26 years) (sex/gender was not self-identified but determined by the experimenter). Prior to the experiment, the Freiburg Visual Acuity test^3^ was administered, and only those who reached a score of at least 1.0 binocularly (corresponding to normal or corrected-to-normal vision) were admitted to the main experiment. Participants signed an informed consent form in advance of the study and received monetary compensation (20 Swiss francs per hour) upon its completion. The experiments and procedures took place in accordance with the Declaration of Helsinki, except for preregistration, and were approved by the local ethics committee (Commission cantonale d’ethique de la recherche sur l’etre humain) of canton Vaud in Switzerland. The experiments and analyses were not preregistered.

### Apparatus

Stimuli were displayed on a BenQ XL2540 LCD monitor with a resolution of 1920 × 1080 pixels, a screen size of 24.5”, and a refresh rate of 240 Hz. Stimuli were generated using MATLAB R2013a (MathWorks Inc., Natick, MA, USA) and Psychtoolbox^4^. Participants were seated 2.5 meters away from the screen, in a dimly lit room. Stimuli were white (100 cd/m²), presented on a black background with a luminance of 1 cd/m².

### Stimuli

Following a fixation dot presented for 0.5 s, and a subsequent blank screen lasting for 0.5 s, the standard SQM stimulus sequence commenced with the presentation of a central line consisting of an upper and a lower segment, each of 26.5’ (arcmin) length and vertically separated by a gap of 2.3’. This central line was then followed by pairs of flanking lines—of the same length and vertical separation as the central line—appearing further and further away from the center. The individual lines had a width of 1.2’ and were horizontally separated by 3.5’. Each line was presented for 20.8 ms. The interstimulus interval (ISI) between the central and first flanking line was 29.2 ms, and the ISI between each of the subsequent flanking lines was 20.8 ms. The stimulus sequence comprised a total of one central and 14 flanking lines with the last flanking line being presented at 590.8 ms. With this stimulus sequence, two motion streams are perceived as diverging from the center towards the periphery.

Participants were asked to covertly attend to one of the streams (in the experiments reported here, the right stream). In the standard SQM configuration, the central line presented at frame 0 was offset (i.e., the lower segment of the line was shifted towards the right or the left, relative to the upper segment; referred to as ‘vernier’ offset or ‘V’), and one of the later lines in the attended stream (whose position was randomly determined to be between the 7^th^ and 13^th^ frame) was offset in the opposite direction (referred to as ‘anti-vernier’ or ‘AV’). Observers perceive only one integrated offset, independent of the number of offsets presented, i.e., observers cannot respond to a single offset and offsets integrate mandatorily^2^. The observers‘ task was to indicate whether they perceived a left or a right offset. Following the completion of the stimulus stream presentation, participants had 3 s to respond by clicking one of two hand-held buttons. Auditory feedback about the correctness of the response was provided in the calibration phase (see the section on calibration below) but not during the main experiment. The inter-trial interval was 0.5 s. The different stimulus configurations used in the three experiments reported in this paper are illustrated in Fig. 2.

In experiment 1, the standard condition is identical to the stimulus configuration introduced above. In the split-vernier condition, three verniers offset in the same direction were presented at frames 0, 3, and 6. The vernier offset sizes at positions 0, 3, and 6 were adjusted for each observer so that performance was equal to the single vernier in the baseline condition (see section on offset calibration).

In the high-load condition of experiment 2, an additional anti-vernier offset was presented at frame 4, and an additional vernier offset was presented at frame 5. This additional pair of anti-vernier and subsequent vernier cancels itself out, i.e., performance was aimed to be similar to the standard condition, but was added to increase the overall processing load.

In experiment 3, the standard condition is identical to the standard condition introduced above, except that a total of 15, instead of 14, pairs of flanking lines were presented and that positions 8 to 13 were sampled for the placement of the anti-vernier (instead of positions 7 to 13). In the short-ISI condition, the ISI was reduced from 20.8 ms to 4.2 ms (but kept at 29.2 ms for the ISI between the central and first flanking line, to keep the metacontrast masking) and the individual lines were horizontally separated by 140, instead of 210, arcsec. The number of flanking lines was increased to 24, and the anti-vernier was randomly presented at positions between 13 and 21.

### Offset size calibration

As detailed above, in the standard SQM condition, a vernier offset is presented at the central line and an anti-vernier offset presented at a later flanking line. To ensure that the individual contributions of these two offsets are perceptually comparable, their offset sizes (i.e., the extent of the horizontal displacement between their top and bottom segment) were individually calibrated, prior to the start of the main experiment. To this end, sequences with a single offset at a given position were presented, and a parameter estimation by sequential testing (PEST) procedure^5^ was used to adaptively determine the offset sizes required to yield around 70% to 80% performance. Given inter-individual differences, this process was carried out for each participant individually. Results of pilot studies suggested that the offset sizes, which are required to reach a certain level of performance, are comparable across the later flanking positions at which anti-vernier offsets would appear (i.e., positions 7-13 in the standard SQM condition). Thus, not all of these positions were calibrated individually. Instead, the offset at frame 10 (i.e., the middle of 7-13) was calibrated and used as a proxy for all later offset positions (i.e., positions 7-13 in the standard condition).

In experiment 1, in the standard condition, offset sizes were calibrated for frames 0 and 10, respectively. In the split-vernier condition, instead of calibrating the offset size at frame 0, the offset sizes were calibrated simultaneously for frames 0, 3, and 6, to ensure that the three offsets appearing together would have the same perceptual strength as the single offset in the standard condition. In both conditions of experiment 2, offset sizes were calibrated for frames 0 and 10, respectively. The offset sizes for the additional pair of anti-vernier and vernier offsets, appearing at positions 4 and 5, were determined by taking the mean of the two calibrated offset sizes for positions 0 and 10. In the standard condition of experiment 3, similar to experiments 1 and 2, frames 0 and 10 were calibrated, respectively. In the short-ISI condition of experiment 3, frames 0 and 17 were calibrated, respectively, with frame 17 chosen to be in the middle of the to be sampled anti-vernier range from position 13 to 21. This range was defined in order to cover temporal coordinates similar to those in the standard condition.

Means and standard deviations of the calibrated offset values across individual observers can be found in Table 1.

**Table 1:** Calibrated offset values.

Experiment, condition, and vernier position	Offset mean (std)
Experiment 1:	
Standard condition: Central vernier (pos. 0)	106.8 (36.1)
Both conditions: Flanking vernier (pos. 10)	61.8 (22.7)
Split-vernier condition: Split central verniers (pos. 0 + 3 + 6)	51.5 (15.9) each
Experiment 2:	
Both conditions: Central vernier (pos. 0)	136.4 (39.4)
Both conditions: Flanking vernier (pos. 10)	65.7 (18.3)
Experiment 3:	
Standard condition: Central vernier (pos. 0)	96.7 (38.2)
Standard condition: Flanking vernier (pos. 10)	51.7 (17.3)
Short-ISI condition: Central vernier (pos. 0)	82.1 (28.9)
Short-ISI condition: Flanking vernier (pos. 17)	68.7 (18.2)

Means and standard deviations of the calibrated offset values (given in arcseconds) for each calibrated offset position for each of the three experiments.

### Experimental procedure

In experiments 1 and 2, participants completed 8 blocks of 84 trials each, for each of the two conditions. In each block, the position at which the anti-vernier in the stimulus sequence appeared was randomly determined in the given range of positions (frames 7–13), while ensuring that each position appeared equally often (here, 12 times each). In experiment 3, participants completed 6 blocks of 84 trials each in the normal-ISI condition (in which across 6 different AV positions were sampled) and 9 blocks of 81 trials each in the short-ISI condition (in which across 9 different AV positions were sampled), to ensure that each position in each condition was repeated at least 80 times. The randomization was applied in order to reduce the potential impact of effects such as fatigue on our results.

### Data analysis

For each experiment and condition, we extracted the anti-vernier dominance level (i.e., the fraction of responses that were in accordance with the direction of the anti-vernier offset) as a function of the position at which the anti-vernier appeared. If the anti-vernier is presented early and the vernier and anti-vernier offsets fall into the same integration window, the anti-vernier dominance would be expected to be close to 50%^1,2^. As the anti-vernier is presented at later and later positions and starting to fall into the next integration window, anti-vernier dominance is expected to increase, finally converging to the calibration level (approximately 75%). If an experimental manipulation were to prolong the duration of the integration window, the slope of the anti-vernier dominance as a function of anti-vernier position would be expected to be less steep. To compare these differences in slope across two experimental conditions, the two resulting data were normalized with regard to the dominance level observed at the earliest measured anti-vernier position (typically, position 7), where full integration occurred^2^.

Note that trials in which reaction times were below 800 ms (i.e., approx. 200 ms following the end of the motion stream) were rejected, as they are likely to have occurred before the end of the stimulus sequence was fully perceived. This procedure did not change the results strongly. The data distribution was assumed to be normal but this was not formally tested. Data analysis was carried out using MATLAB R2022b (MathWorks Inc., Natick, MA, USA).

For the Bayes factor analysis reported in experiment 1 (for MATLAB package, see^6^), the default prior (Cauchy width = 0.707) was chosen, revealing a moderate Bayes factor of 0.298. This result generalizes well across priors: a wide prior (Cauchy width = 1) would yield a moderate Bayes factor of 0.223 and an ultrawide prior (Cauchy width = 1.414) a moderate Bayes factor of 0.164. This analysis of prior dependence was carried out using JASP (version 0.17.2.1).

### Power analysis

The SQM^1^ has a large effect size, with a Cohen’s d of usually around 1.5 or greater^2,7^. With this effect size, a power analysis, computed with the G*Power software, revealed that a modest sample size of less than 10 participants would be required for achieving a power of 90%. To be on the safe side, we here recruited more than 10 participants for each experiment.

### Reporting summary

Further information on research design is available in the Nature Portfolio Reporting Summary linked to this article.

## Results

### Window duration is not dependent on the timing of stimulus evidence presentation

**Fig. 3:**
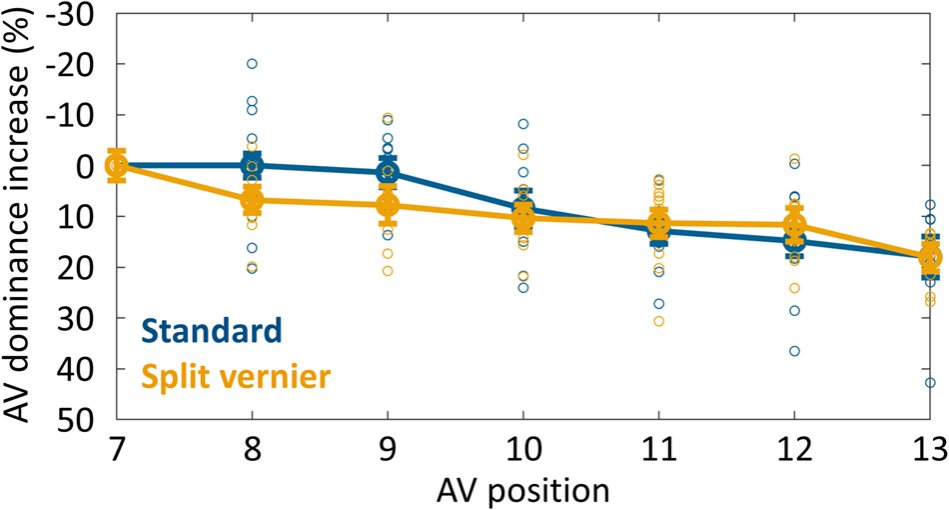
Results of experiment 1. Increase in anti-vernier dominance relative to the anti-vernier dominance of frame 7, for the standard (blue line) and split-vernier (yellow line) condition. Performance in the two conditions is roughly the same. Error bars depict standard errors; circles depict individual participants’ data points. There is moderate evidence in favor of an absence of an effect of difference between both conditions (Bayes factor = 0.298, median of effect size = 0.015; 95% CI of effect size = [−0.512, 0.544] in a two-tailed paired-sample comparison of slopes; *n* = 11 independent participants).

In experiment 1, either a large offset at the central line (standard condition) or 3 smaller offsets at lines 0, 3, and 6, all in the same direction (split-vernier condition), were presented (see Fig. 2 for illustration). Classical models of decision-making, in which sensory evidence is integrated until a decision threshold is reached and a motor response elicited, would predict that integration windows in the split-vernier condition may be longer, considering that evidence about the offset to be reported is presented later, as the vernier offset information is dispersed along the motion stream instead of being available right from the beginning. As can be seen in Fig. 3, if any, the increase in anti-vernier dominance would rather show the opposite effect. We fitted regression lines (fitting only the slope; keeping the intercept constant, so as to enforce an AV dominance increase of 0 at the first AV position) to the individual participants’ data, separately for both conditions, and found moderate evidence in favor of an absence of the effect (Bayes factor = 0.298, median of effect size = 0.015, 95% CI of effect size = [-0.512, 0.544] in a two-tailed paired-sample comparison of slopes; *n* = 11). Hence, evidence dispersed along the motion stream, rather than presented right from the beginning, does not prolong integration.

### Higher processing load extends window duration

**Fig. 4:**
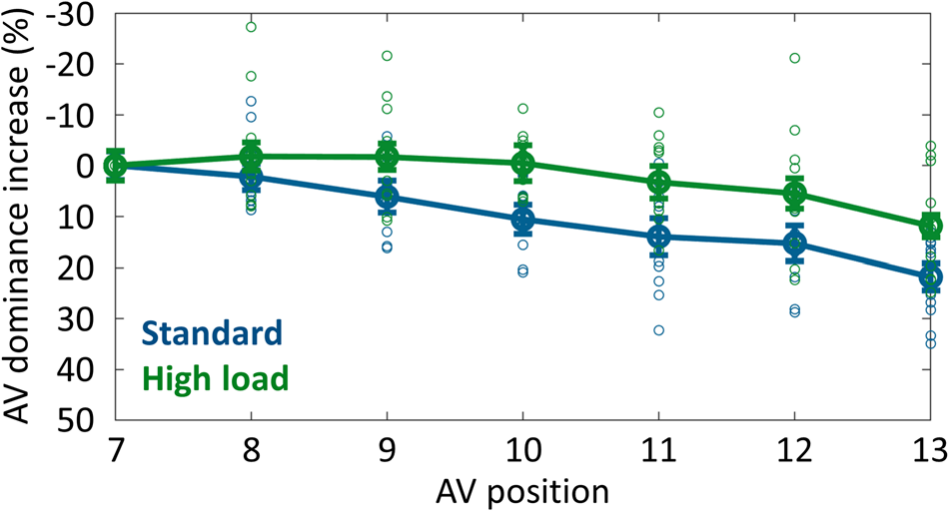
Results of experiment 2. Increase in anti-vernier dominance for the standard (blue line) and high-load (green line) condition, relative to the anti-vernier dominance of frame 7. Error bars depict standard errors; circles depict individual participants’ datapoints. The two conditions differed significantly in their slope (*t*(10) = 3.10, *p* = .011, Cohen’s *d* = 1.250, CI = [0.633, 3.860] in two-tailed paired-sample t-test; *n* = 11 independent participants).

Relative to the standard condition, two further offsets at two additional lines were added in the high-load condition, which are expected to consciously cancel each other out but increase the overall unconscious processing load, due to there being more elements to be processed. As depicted in Fig. 4, the integration profiles across the two conditions differed significantly in their slope (*t*(10) = 3.10, *p* = .011, Cohen’s *d* = 1.250, 95% CI = [0.633, 3.860] in two-tailed paired-sample t-test for individual participants’ lines of best fits for the two conditions; *n* = 11), indicating that higher processing load extends the duration of temporal integration windows.

### Integration windows depend on absolute time, rather than on the number of lines

In experiment 3, the inter-stimulus interval was reduced from 20.8 ms to 4.2 ms, leading to the presentation of more lines in the same amount of time. If, in the standard SQM setting, integration lasts for around 10 frames, corresponding to 425 ms, will integration in the short-ISI condition last for around 10 frames, corresponding to 275 ms, or will it last for around 16 frames, corresponding to 425 ms? In other words, is the extent of integration, as measured in the SQM, a temporal measure or does it depend on the number of discrete elements presented?

**Fig. 5:**
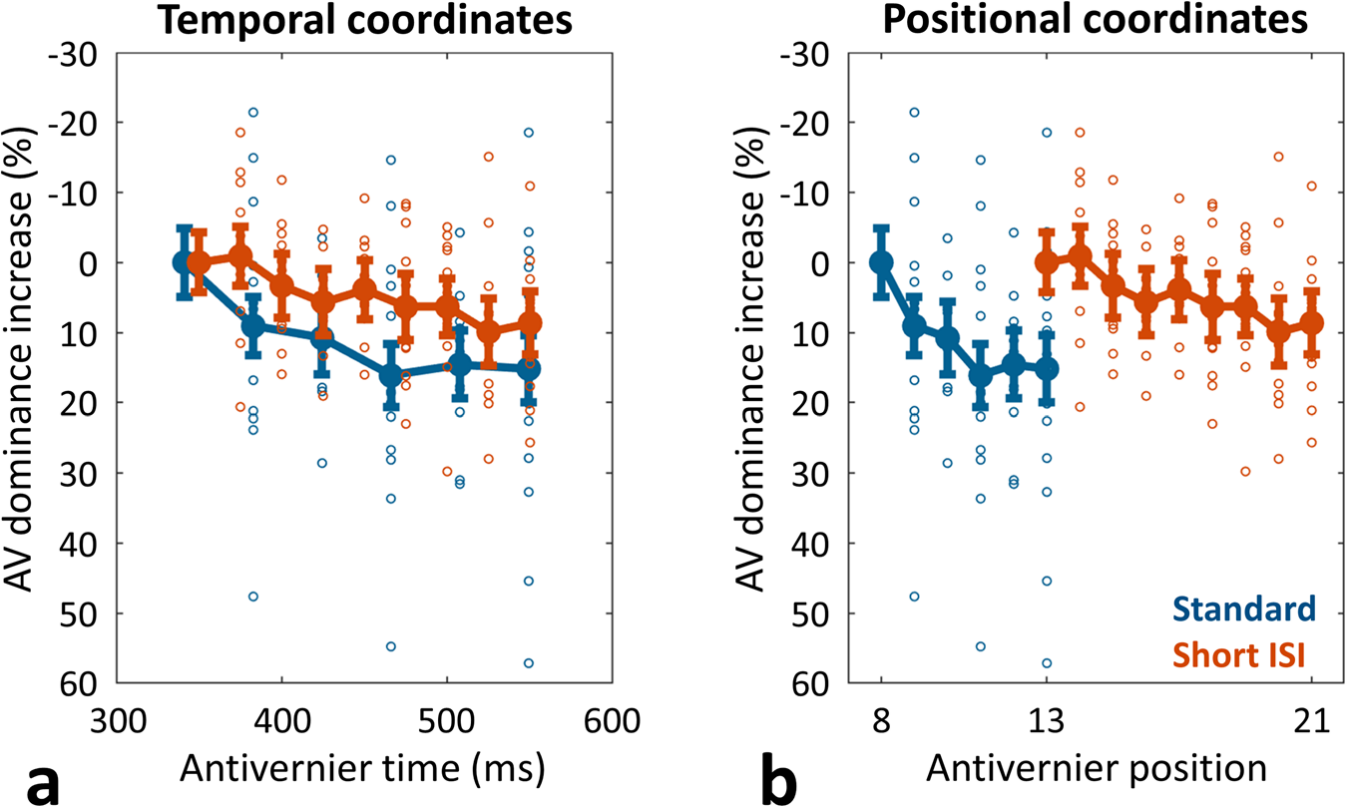
Results of experiment 3. Increase in anti-vernier dominance across anti-vernier time/position, relative to the anti-vernier dominance obtained at the first measured time point/position, for the standard condition (blue line) and the short-ISI condition (red line), when plotted in a temporal coordinate system **a** or a positional coordinate system **b**. Error bars depict standard errors; circles depict individual participants’ data points. The two curves depicted in temporal coordinates are significantly more aligned than those depicted in positional coordinates (*t*(14) = 2.98, *p* = .010, Cohen’s *d* = 1.07, 95% CI = [4.233, 26.098], in a two-tailed paired-sample t-test; *n* = 15 independent participants).

The left panel of Fig. 5 depicts the two conditions’ integration profiles when they are aligned with regard to the time at which the anti-vernier is presented. In contrast, the right panel of Fig. 5 depicts the integration profiles when they are aligned with regard to the position at which the anti-vernier is presented. The two curves depicted in temporal coordinates are significantly more aligned than those depicted in positional coordinates (*t*(14) = 2.98, *p* = 0.010, Cohen’s *d* = 1.07, 95% CI = [4.233, 26.098] in a two-tailed paired-sample t-test comparing differences between unnormalized AV dominance at position 13 (presented at 350 ms) of the short-ISI condition [mean = 58.9%] with the temporal (position 8, at ca. 350 ms) vs. the positional (position 13) equivalent in the standard condition [means = 57.2% and 72.3%, respectively]; *n* = 15). Thus, the absolute time, rather than the number of lines, determines window duration.

## Discussion

### Substantially-delayed conscious perception

Conscious perception is substantially delayed, preceded by extended periods of unconscious processing^1,2,9,10^ (but see discussion^11–13^). These periods are crucial, for example, for detecting and integrating motion signals, solving many of the ill-posed problems of vision, and integrating information in low-contrast scenarios. In this paper, we examined how long these periods of temporal integration last and what factors determine them. The SQM has turned out to be a versatile tool for this purpose as its mandatory integration allows measuring how information at specific time points affects integration^1,2,7,14–18^.

### Processing load, and not stimulus evidence, determines integration duration

Here, we have shown that the duration of unconscious feature integration is not significantly affected by the evidence presented. Specifically, the duration of the integration window did not differ depending on whether strong evidence was presented right from the beginning or was dispersed along the motion stream. This result is surprising considering that in classical models of decision making, the stronger the evidence, the quicker a decision is reached and a motor response elicited^19^. We would have expected a similar result for the duration of a window of integration. In this respect, our results suggest that classical decision-making and the duration of integration are only weakly linked, subserving potentially distinct neural processes (see also the work of Rüter and colleagues^20,21^).

Instead of evidence-based integration termination, our second experiment revealed that processing load determines integration duration. When adding additional vernier and anti-vernier offsets that canceled each other out but increased the overall processing load, integration lasted longer. This result, we propose, can be accounted for by the brain continuously monitoring, and controlling for, the difficulty of tasks. This appears intuitively plausible: in a challenging perceptual scenario, such as a low-contrast situation, one benefits from extended integration windows. In our experiment specifically, the repetitive and block-wise presentation of SQM stimulus streams may have also helped to form expectations prior to the onset of a given trial about task difficulty. We believe that this proposal opens a new avenue for future research, not just focused on the brain’s processing but also on the monitoring of its own processing.

### Integration windows depend on absolute time, not on the number of lines

Further, as revealed in our third experiment, it is the absolute time (i.e., a temporal measure quantifiable in milliseconds), and not the number of lines (i.e., a measure of the number of discrete elements in the motion stream), that determines the extent of unconscious feature integration. Interestingly, this finding differs from those that have been reported in some other paradigms: for instance, studies of semantic monitoring of rapidly-presented word lists have revealed that recovery from shift costs (i.e., recovery from performance drops following a cue to shift attention) occurs as a function of the number of presented items, rather than the absolute time, following the shift cue^22,23^.

### Minimum integration periods

While our results reveal that an increase in the processing load extends the integration time, it is important to note that there appears to be a minimum integration period with the SQM paradigm, longer than around 350 ms. These long periods even exist when the stimuli themselves are presented for much shorter durations^18^. Interestingly, the long periods of unconscious integration observed in the SQM mirror results reported in other paradigms. For instance, it was shown that integration in RSVP paradigms can last longer than 200 ms^24^, and that the presentation of a cue that appeared up to 400 ms following a visual stimulus changed the perception of the latter^25,26^. Further, it is important to note that even if experiments reveal short effects of integration, conscious visual perception may nevertheless occur at a later point in time.

### Limitations

While we here have examined the role of stimulus evidence, processing load, and the number of elements vs. absolute time on the duration of unconscious visual integration, there are many more relevant features that will need to be examined in future work to gain a more wholesome understanding of the dynamics underlying temporal integration mechanisms.

## Conclusion

Taken together, based on the experiments with the SQM that we report in this paper, we propose that the extent of discrete windows of unconscious feature integration depends mainly on the processing load, not on the stimulus evidence, and can be measured in absolute time rather than the number of elements presented in a motion stream.

### Supplementary information

Peer Review

Reporting Summary

## Data Availability

The data underlying this study is available at 10.5281/zenodo.8113855^8^. Please contact the lead author (Lukas Vogelsang) in case of any questions as well as for additional data.

## Code availability

The analysis code is available at 10.5281/zenodo.8113855^8^.
