# Supplementary material for: Processing load, and not stimulus evidence, determines the duration of unconscious visual feature integration
Source: Commun Psychol. 2023 Aug 8;1:8. doi: 10.1038/s44271-023-00011-2 (PMC11041769; doi:10.1038/s44271-023-00011-2)
Supplement: Supplementary file 2 — Reporting Summary [file 44271_2023_11_MOESM2_ESM.pdf]

nature portfolio

Corresponding author(s): Lukas Vogelsang
Last updated by author(s): Jul 4, 2023

Reporting Summary

Nature Portfolio wishes to improve the reproducibility of the work that we publish. This form provides structure for consistency and transparency in reporting. For further information on Nature Portfolio policies, see our Editorial Policies and the Editorial Policy Checklist.

Statistics

For all statistical analyses, confirm that the following items are present in the figure legend, table legend, main text, or Methods section.

n/a	Confirmed	
☐	☒	The exact sample size (*n*) for each experimental group/condition, given as a discrete number and unit of measurement
☐	☒	A statement on whether measurements were taken from distinct samples or whether the same sample was measured repeatedly
☐	☒	The statistical test(s) used AND whether they are one- or two-sided<br>*Only common tests should be described solely by name; describe more complex techniques in the Methods section.*
☒	☐	A description of all covariates tested
☒	☐	A description of any assumptions or corrections, such as tests of normality and adjustment for multiple comparisons
☐	☒	A full description of the statistical parameters including central tendency (e.g. means) or other basic estimates (e.g. regression coefficient) AND variation (e.g. standard deviation) or associated estimates of uncertainty (e.g. confidence intervals)
☐	☒	For null hypothesis testing, the test statistic (e.g. *F*, *t*, *r*) with confidence intervals, effect sizes, degrees of freedom and *P* value noted<br>*Give P values as exact values whenever suitable.*
☐	☒	For Bayesian analysis, information on the choice of priors and Markov chain Monte Carlo settings
☒	☐	For hierarchical and complex designs, identification of the appropriate level for tests and full reporting of outcomes
☐	☒	Estimates of effect sizes (e.g. Cohen's *d*, Pearson's *r*), indicating how they were calculated

Our web collection on statistics for biologists contains articles on many of the points above.

Software and code

Policy information about availability of computer code

Data collection	The experiment was programmed using MATLAB R2013a (MathWorks Inc., Natick, MA, USA) and Psychtoolbox (Brainard, 1997).
Data analysis	Data analysis was carried out using MATLAB R2022b (MathWorks Inc., Natick, MA, USA).

For manuscripts utilizing custom algorithms or software that are central to the research but not yet described in published literature, software must be made available to editors and reviewers. We strongly encourage code deposition in a community repository (e.g. GitHub). See the Nature Portfolio guidelines for submitting code & software for further information.

Data

Policy information about availability of data

All manuscripts must include a data availability statement. This statement should provide the following information, where applicable:

- Accession codes, unique identifiers, or web links for publicly available datasets
- A description of any restrictions on data availability
- For clinical datasets or third party data, please ensure that the statement adheres to our policy

The data and analysis code underlying the figures of the manuscript are available in a Zenodo repository, as detailed in the data/code availability statement.

Human research participants

Policy information about studies involving human research participants and Sex and Gender in Research.

Reporting on sex and gender	We recruited 3 female and 8 male participants in experiment 1, 2 female and 9 male participants in experiment 2, and 5 female and 10 male participants in experiment 3. We did not analyse the data as a function of sex/gender, as we did not intend to test sex/gender-specific differences but general perceptual hypotheses. Further, for a given experiment, participants were not divided into separate groups but participated in all conditions. Thus, there was no need for specific balancing.
Population characteristics	The age range of participants was 19-24 years for experiment 1, 19-26 years for experiment 2, and 18-26 years for experiment 3. All participants had normal or corrected-to-normal vision.
Recruitment	Participants were recruited from the École Polytechnique Fédérale de Lausanne and the University of Lausanne in Switzerland.
Ethics oversight	This study was approved by the local ethics committee (Commission cantonale d'ethique de la recherche sur l'etre humain) of canton Vaud in Switzerland.

Note that full information on the approval of the study protocol must also be provided in the manuscript.

Field-specific reporting

Please select the one below that is the best fit for your research. If you are not sure, read the appropriate sections before making your selection.

☒ Life sciences ☐ Behavioural & social sciences ☐ Ecological, evolutionary & environmental sciences

For a reference copy of the document with all sections, see nature.com/documents/nr-reporting-summary-flat.pdf

Life sciences study design

All studies must disclose on these points even when the disclosure is negative.

Sample size	The effect size of the experimental paradigm (SQM) has a Cohen's d greater than or equal to 1.5. Therefore, a modest sample size of less than 10 participants would be required for achieving a power of 90%. To be on the safe side, we here recruited more than 10 participants for each experiment.
Data exclusions	Participants were only excluded if they failed the initial visual acuity test or if not all parts of the experiment were successfully completed/ recorded. Individual trials were only excluded if the reaction time was too fast. All of this is detailed in the methods section of the manuscript.
Replication	We did not actively try to replicate the three experiments presented in this paper, but the data we obtained are generally consistent across experiments and also with previous papers using the SQM experimental paradigm.
Randomization	For a given experiment, participants were not divided into groups but participated in all conditions. Thus, randomized allocation of participants into different experimental groups is not relevant to this study.
Blinding	For a given experiment, participants were not divided into groups but participated in all conditions. Thus, blinding is not relevant to this study.

Reporting for specific materials, systems and methods

We require information from authors about some types of materials, experimental systems and methods used in many studies. Here, indicate whether each material, system or method listed is relevant to your study. If you are not sure if a list item applies to your research, read the appropriate section before selecting a response.

Materials & experimental systems

n/a	Involved in the study	
☒	☐	Antibodies
☒	☐	Eukaryotic cell lines
☒	☐	Palaeontology and archaeology
☒	☐	Animals and other organisms
☒	☐	Clinical data
☒	☐	Dual use research of concern

Methods

n/a	Involved in the study	
☒	☐	ChIP-seq
☒	☐	Flow cytometry
☒	☐	MRI-based neuroimaging
