# Supplementary material for: Processing load, and not stimulus evidence, determines the duration of unconscious visual feature integration
Source: Commun Psychol. 2023 Aug 8;1:8. doi: 10.1038/s44271-023-00011-2 (PMC11041769; doi:10.1038/s44271-023-00011-2)
Supplement: Supplementary file 1 — Peer Review [file 44271_2023_11_MOESM1_ESM.pdf]

nature portfolio

Peer Review File

Processing load, and not stimulus evidence, determines the duration of unconscious visual feature integration

Open Access This file is licensed under a Creative Commons Attribution 4.0 International License, which permits use, sharing, adaptation, distribution and reproduction in any medium or format, as long as you give appropriate credit to the original author(s) and the source, provide a link to the Creative Commons license, and indicate if changes were made. In the cases where the authors are anonymous, such as is the case for the reports of anonymous peer reviewers, author attribution should be to 'Anonymous Referee' followed by a clear attribution to the source work. The images or other third party material in this file are included in the article's Creative Commons license, unless indicated otherwise in a credit line to the material. If material is not included in the article's Creative Commons license and your intended use is not permitted by statutory regulation or exceeds the permitted use, you will need to obtain permission directly from the copyright holder. To view a copy of this license, visit http://creativecommons.org/licenses/by/4.0/.

Web links to the author's journal account have been redacted from the decision letters as indicated to maintain confidentiality

11th Jan 23

Dear Mr Vogelsang,

Thank you for transferring your manuscript titled "Processing load, and not stimulus evidence, determines the duration of unconscious visual feature integration" to Communications Psychology.

We are looking forward to sending your manuscript to peer review. However, the work does not yet fully comply with our statistics guidelines, and we would like to ask you to revise it accordingly before we send it to the reviewers.

In particular, the manuscript contains a number of statements where the absence of evidence for an effect (a non-significant result in NHSTs) is taken as evidence of the absence of an effect (or difference). These instances would need to be accompanied by equivalence tests of Bayesian statistics that support the interpretation that conditions do not differ. You can read more about our statistics requirements for null results here:
https://www.nature.com/commspsychol/submit/submission-guidelines#statistical-guidelines

Marginally significant statistical results can be mentioned as such, but should not be discussed as theoretically informative for the hypotheses tested.

We take the unusual course of action to return manuscripts to authors prior to peer review when we think it will avoid unproductive rounds of review that ultimately reduce the chances of the manuscript obtaining a fair and objective evaluation. If you are able to adequately respond to these concerns, we would be happy to send your manuscript out for review.

We hope to receive your revised version as soon as possible. If you anticipate a delay of more than three months, however, please let us know. We will be happy to consider your revision so long as nothing similar has been accepted for publication at Communications Psychology or published elsewhere.

If you are not interested in submitting a suitably revised manuscript in the future please let me know immediately so we can close your file. If you have any questions, please contact me.

Please use the link below to submit a suitably revised manuscript and updated response to referees when they are ready.

[link redacted]
** This url links to your confidential home page and associated information about manuscripts you may have submitted or be reviewing for us. If you wish to forward this email to co-authors, please delete the link to your homepage first **

In the meantime, I regret that we remain unable to publish your current manuscript in Communications Psychology. We would naturally understand, therefore, if you decided to submit your manuscript elsewhere.

Best regards,

Antonia Eisenkoeck

Antonia Eisenkoeck
Senior Editor
Communications Psychology

27th Feb 23

Dear Mr Vogelsang,

Thank you for your patience during the peer-review process. Your manuscript titled "Processing load, and not stimulus evidence, determines the duration of unconscious visual feature integration" has now been seen by 2 reviewers, whose comments are appended below. You will see that they find your work of some potential interest. However, they have raised quite substantial concerns that must be addressed. In light of these comments, we cannot accept the manuscript for publication, but would be interested in considering a revised version that fully addresses these serious concerns by providing additional data to support all conclusions. As per journal policy, we require at least moderate evidence from Bayesian tests to support any conclusions; interpretations based on anecdotal evidence need to be substantiated by further data, or removed.

We hope you will find the Reviewers' comments useful as you decide how to proceed. Should additional work allow you to address these criticisms, we would be happy to look at a substantially revised manuscript. If you choose to take up this option, please highlight all changes in the manuscript text file, and provide a detailed point-by-point reply to the reviewers.

Please bear in mind that we will be reluctant to approach the reviewers again in the absence of substantial revisions.

Editorially, we would like to ask you to implement appropriate language to reflect the strength of evidence. For this purpose, please use Lee, M.D., & Wagenmakers, E.-J. (2013). Bayesian cognitive modeling: A practical course. Cambridge University Press, as a basis for your interpretation of the Bayes Factors. If you do not have access to the publication, you can refer to: Schönbrodt, F.D., Wagenmakers, E. Bayes factor design analysis: Planning for compelling evidence. Psychon Bull Rev 25, 128–142 (2018) https://rdcu.be/b6uOC.

If the revision process takes significantly longer than five months, we will be happy to reconsider your paper at a later date, provided it still presents a significant contribution to the literature at that stage.

We understand that due to the current global situation, the time required for revision may be longer than usual. We would appreciate it if you could keep us informed about an estimated timescale for resubmission, to facilitate our planning. Of course, if you are unable to estimate, we are happy to accommodate necessary extensions nevertheless.

We are committed to providing a fair and constructive peer-review process. Please do not hesitate to contact us if you wish to discuss the revision in more detail.

Please use the following link to submit your revised manuscript, point-by-point response to the Reviewers' comments with a list of your changes to the manuscript text (which should be in a separate document to any cover letter) and any completed checklist:

[link redacted]

** This url links to your confidential home page and associated information about manuscripts you may have submitted or be reviewing for us. If you wish to forward this email to co-authors, please delete the link to your homepage first **

Please do not hesitate to contact me if you have any questions or would like to discuss the required revisions further. Thank you for the opportunity to review your work.

Best regards,

Antonia Eisenkoeck

Antonia Eisenkoeck
Senior Editor
Communications Psychology

EDITORIAL POLICIES AND FORMATTING

We ask that you ensure your manuscript complies with our editorial policies. Please ensure that the following formatting requirements are met, and any checklist relevant to your research is completed and uploaded as a Related Manuscript file type with the revised article.

Editorial Policy: Policy requirements (Download the link to your computer as a PDF.)

Furthermore, please align your manuscript with our format requirements, which are summarized on the following checklist:
Communications Psychology formatting checklist

and also in our style and formatting guide Communications Psychology formatting guide .

* TRANSPARENT PEER REVIEW: Communications Psychology uses a transparent peer review system. This means that we publish the editorial decision letters including Reviewers' comments to the authors and the author rebuttal letters online as a supplementary peer review file. However, on author request, confidential information and data can be removed from the published reviewer reports and rebuttal letters prior to publication. If your manuscript has been previously reviewed at another journal, those Reviewers' comments would not form part of the published peer review file.

* CODE AVAILABILITY: All Communications Psychology manuscripts must include a section titled "Code Availability" at the end of the methods section. In the event of publication, we require that the custom analysis code supporting your conclusions is made available in a publicly accessible repository; at publication, we ask you to choose a repository that provides a DOI for the code; the link to the repository and the DOI will need to be included in the Code Availability statement. Publication as Supplementary Information will not suffice. We ask you to prepare code at this stage,

to avoid delays later on in the process.

* DATA AVAILABILITY:
All Communications Psychology research manuscripts must include a section titled "Data Availability" at the end of the Methods section or main text (if no Methods). More information on this policy, is available at http://www.nature.com/authors/policies/data/data-availability-statements-data-citations.pdf.

At a minimum the Data availability statement must explain how the data can be obtained and whether there are any restrictions on data sharing. Communications Psychology strongly endorses open sharing of da. If you do make your data openly available, please include in the statement:
- Unique identifiers (such as DOIs and hyperlinks for datasets in public repositories)
- Accession codes where appropriate
- If applicable, a statement regarding data available with restrictions
- If a dataset has a Digital Object Identifier (DOI) as its unique identifier, we strongly encourage including this in the Reference list and citing the dataset in the Data Availability Statement.

We recommend submitting the data to discipline-specific, community-recognized repositories, where possible and a list of recommended repositories is provided at http://www.nature.com/sdata/policies/repositories.

If a community resource is unavailable, data can be submitted to generalist repositories such as figshare or Dryad Digital Repository. Please provide a unique identifier for the data (for example a DOI or a permanent URL) in the data availability statement, if possible. If the repository does not provide identifiers, we encourage authors to supply the search terms that will return the data. For data that have been obtained from publicly available sources, please provide a URL and the specific data product name in the data availability statement. Data with a DOI should be further cited in the methods reference section.

Please refer to our data policies at http://www.nature.com/authors/policies/availability.html.

REVIEWER EXPERTISE:
Reviewer #1: visual processing, data analysis
Reviewer #2: visual processing

Reviewer #1 (Remarks to the Author):

There is now convincing evidence that basic visual features are integrated over a temporal window before being consciously perceived. What is not known, however, is when this temporal window terminates. Using a clever experimental design, the authors show that the duration of the temporal window can be increased when there are more events to process. However, that duration does not seem to be affected by the way the event is presented, namely if it is shown all at once or

progressively. These results are very important for our understanding of visual perception, not just time perception. I only have one comment.

It seems that the so-called "high-load" condition was run with the sequence V1-V2-AV (where "V" stands for Vernier (1st and 2nd presentations) and "AV" for anti-Vernier; see line 232). The authors claim that the last pair V2-AV "cancelled itself out" (line 234). However, isn't it possible that V2 was integrated with whatever was presented so far, i.e. V1, thus producing a very salient Vernier effect that is then reduced by the presentation of AV? Or is it the temporal proximity of V2 and AV that makes this pair cancelling itself out? If the authors are correct, then they should obtain the same high-load effect with the sequence V1-AV-V2, where the pair AV-V2 would cancel itself out. If the alternative interpretation above is plausible, then one might expect a disappearance of the high-load effect, because it would be V1 and AV that cancel itself out. Maybe the authors can discuss this point on the importance (or lack of) of the order of events.

Minor comment:
- In the offset size calibration (lines 246ff), I think it would be informative to report the calibrated offsets for the different experiments, maybe the mean across participants or for one representative participant. In particular, this information would be valuable in the "split-Vernier" condition where the offset is, by definition, split across 3 positions, so that one can compare the sum of these offsets to the full offset in the "standard" condition.

Reviewer #2 (Remarks to the Author):

Review of Vogelsang, Drissi-Daoudi, & Herzog "Processing load, and not stimulus evidence, determines the duration of unconscious visual feature integration"

Comments
The authors present a set of novel experiments based on an interesting paradigm, which produces potentially interesting findings for a subfield in psychology, as the journal requires. As such, I think the manuscript has merit. The manuscript would benefit from some clarifications/explanations, however, which will hopefully make the impact of the research more clear.

The logic of Exp 1 is not entirely clear to me. In the Split Vernier version, integration is expected to be longer, as "evidence is presented later". Presumably, this refers to the initial verniers. However, the length of the sequence is otherwise unchanged, as is the onset of the final antivernier. Would integration be expected to differ, if observers have to wait equally long for the streams to end?

In Exp 2, the extra verniers at position 4 and 5 are supposed to increase "processing load". First, is there any (prior) evidence that this is the case? Second, although the slopes of the two curves for high and normal load do indeed differ, the final level of antivernier dominance also differs. So is this really a change in the window of integration, or do the extra verniers just prevent the final antivernier from having its normal impact on the percept? Similarly, in Exp 3, the short and normal ISI conditions do not converge on the same level of antivernier dominance. I understand that the authors want to assess just the slope of the dominance curve over time, but I wonder whether this is an unbiased measure of the range differs between conditions.

Minor
The SQM paradigm, and the subtle variations employed in the present paper are difficult to follow for the non-expert. Perhaps combining Fig. 1 and 5 might help here.

The results figures could be more informative (Fig. 2, 3, and 4). As it is only group means and standard error (?) are shown, not individual data points, which would provide a more comprehensive summary of the results.

There is no mention of publicly sharing the data and analysis scripts. I would recommend the authors to do so.

Reviewer #1 (Remarks to the Author):

There is now convincing evidence that basic visual features are integrated over a temporal window before being consciously perceived. What is not known, however, is when this temporal window terminates. Using a clever experimental design, the authors show that the duration of the temporal window can be increased when there are more events to process. However, that duration does not seem to be affected by the way the event is presented, namely if it is shown all at once or progressively. These results are very important for our understanding of visual perception, not just time perception. I only have one comment.

We are very glad to hear that you find our work to be of interest.

It seems that the so-called “high-load” condition was run with the sequence V1-V2-AV (where “V” stands for Vernier (1st and 2nd presentations) and “AV” for anti-Vernier; see line 232). The authors claim that the last pair V2-AV “cancelled itself out” (line 234). However, isn’t it possible that V2 was integrated with whatever was presented so far, i.e. V1, thus producing a very salient Vernier effect that is then reduced by the presentation of AV? Or is it the temporal proximity of V2 and AV that makes this pair cancelling itself out? If the authors are correct, then they should obtain the same high-load effect with the sequence V1-AV-V2, where the pair AV-V2 would cancel itself out. If the alternative interpretation above is plausible, then one might expect a disappearance of the high-load effect, because it would be V1 and AV that cancel itself out. Maybe the authors can discuss this point on the importance (or lack of) of the order of events.

Many thanks for this important comment, which, in fact, helped us realize that our illustration was not in full accordance with how the experiment was carried out. Instead of vernier-antivernier-antivernier-vernier sequences, we were actually presenting vernier-antivernier-vernier-antivernier sequences, i.e., the sequence you were describing above. We apologize for this error in our initial illustration and have now corrected the illustration and legend accordingly.

Nevertheless, your comment points to an important general question concerning the effect of the ordering of events within a given discrete window of integration. Thus far, we have not found any evidence for such ordering to matter, but we cannot absolutely rule out this possibility.

Minor comment:
- In the offset size calibration (lines 246ff), I think it would be informative to report the calibrated offsets for the different experiments, maybe the mean across participants or for one representative participant. In particular, this information would be valuable in the “split-Vernier” condition where the offset is, by definition, split across 3 positions, so that one can compare the sum of these offsets to the full offset in the “standard” condition.

Many thanks. We have now included the means and standard deviations of the calibrated offsets for each of the positions, conditions, and experiments in the manuscript.

Regarding your specific point about experiment 1, you will see that the mean calibrated offset size of the central vernier (106.8 arcsec) in the standard condition is a little more than twice as large as that of each of the three verniers in the split-vernier condition (51.5 arcsec).

Reviewer #2 (Remarks to the Author):

Review of Vogelsang, Drissi-Daoudi, & Herzog "Processing load, and not stimulus evidence, determines the duration of unconscious visual feature integration"

Comments

The authors present a set of novel experiments based on an interesting paradigm, which produces potentially interesting findings for a subfield in psychology, as the journal requires. As such, I think the manuscript has merit. The manuscript would benefit from some clarifications/explanations, however, which will hopefully make the impact of the research more clear.

We are glad to hear that you find this work to have merit, and would like to thank you for your comments that helped us to significantly improve the manuscript.

The logic of Exp 1 is not entirely clear to me. In the Split Vernier version, integration is expected to be longer, as "evidence is presented later". Presumably, this refers to the initial verniers. However, the length of the sequence is otherwise unchanged, as is the onset of the final antivernier. Would integration be expected to differ, if observers have to wait equally long for the streams to end?

Thank you for pointing out the need for further clarification. As we had stated in the results section, "Classical models of decision making would predict that integration windows in the 'split vernier'-condition may be longer, considering that evidence is presented later." What this relates to is the idea that in such models, sensory evidence is integrated until a decision threshold is reached (and a motor response elicited). Thus, if strong evidence is presented earlier (as is the case in the standard condition, relative to the split-vernier condition), a decision should be reached earlier. However, as described in the manuscript, this is not what we found.

We have rephrased the introductory sentence in the results section as follows (insertions highlighted in **bold**): "Classical models of decision making, **in which sensory evidence is integrated until a decision threshold is reached and a motor response elicited**, would predict that integration windows in the 'split vernier'-condition may be longer, considering that evidence **about the offset to be reported** is presented later**, as the vernier offset information is dispersed along the motion stream instead of being available right from the start**."

In Exp 2, the extra verniers at position 4 and 5 are supposed to increase "processing load". First, is there any (prior) evidence that this is the case?

Thank you for raising this point. While we do not have any prior evidence for the additional verniers at positions 4 and 5 to be increasing the "consciously-perceived processing load" in terms of phenomenology, what we actually meant to express is that it increases the "unconscious processing load", simply due to there being two additional verniers to be processed.

We have rephrased the first sentence relating to this in the results section as follows (insertions in **bold**): "Relative to the standard condition, two further offsets at two additional lines were added, which **are expected to consciously** cancel~~ed~~ each other out but **to** increase~~d~~ the overall **unconscious** processing load**, due to there being more elements to be processed** ('high load'-condition).".

Second, although the slopes of the two curves for high and normal load do indeed differ, the final level of antivernier dominance also differs. So is this really a change in the window of integration, or do the extra verniers just prevent the final antivernier from having its normal impact on the percept?

Similarly, in Exp 3, the short and normal ISI conditions do not converge on the same level of antivernier dominance. I understand that the authors want to assess just the slope of the dominance curve over time, but I wonder whether this is an unbiased measure of the range differs between conditions.

Many thanks for raising this important point. There are two aspects to consider:

First, while we agree that the final level of antivernier dominance differs between the two conditions (considering that we measured the same range of antivernier positions in both conditions but that a full match in terms of antivernier dominance would require later antivernier positions in the high-load, relative to the standard, condition), we strongly believe that it is indicative of a change in the window of integration instead of the extra verniers simply preventing “the final antivernier from having its normal impact on the percept”. The data presented in the manuscript provide counterevidence for the idea that the presentation of the additional antivernier-vernier pair at positions 4 and 5 would, for instance, disrupt and reset the current integration window as, in this case, none of the final antiverniers presented at positions 7 through 13 should be integrated with the initial vernier anymore, and AV dominance should immediately drop from 50% to approximately 25%. Thus, we would expect that when measuring a larger range of antivernier positions, really capturing the end of the integration window also in the high-load condition, we would see both converging to similar dominance levels and thereby more clearly depicting a shift in the window size.

Further, past control data on six participants we had recorded in the context of experiment 2 confirm this intuition. There is a marked difference in vernier dominance (19.6 percent points) when presenting a central vernier together with an antivernier at position 10 vs. presenting a central vernier together with an antivernier at position 18 (see figure below). Thus, we believe that we can, indeed, take the difference in slope as an effective proxy for window shifts.

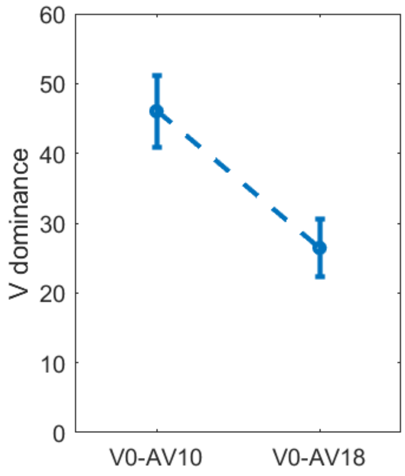

The second question your point raises is whether the specifics of the metric and statistic we have chosen quantitatively captures this proxy well. Here, your comment helped us realize that our previous comparison of regression line slopes (when simultaneously fitting slope AND intercept) was suboptimal, considering that the fitted lines do not necessarily go through an “AV dominance increase” of 0 for the first AV position measured. Thus, we have adjusted the procedure to only fitting the slope (thereby enforcing a y-value of 0 for the first AV position / timepoint measured), and re-computed the statistics accordingly. This did not affect the subsequent interpretation.

Minor

The SQM paradigm, and the subtle variations employed in the present paper are difficult to follow for the non-expert. Perhaps combining Fig. 1 and 5 might help here.

Thank you. We have now placed Fig. 5 immediately after Fig. 1.

The results figures could be more informative (Fig. 2, 3, and 4). As it is only group means and standard error (?) are shown, not individual data points, which would provide a more comprehensive summary of the results.

Thank you for this comment. We have added individual data points to each of the figures and also added to the legend that our error bars depict the standard error.

There is no mention of publicly sharing the data and analysis scripts. I would recommend the authors to do so.

Thank you for this recommendation. We will share the data and analysis scripts required for reproducing our figures publicly, and have added this to our code/data availability statement and reporting summary accordingly.

14th Apr 23

Dear Mr Vogelsang,

Thank you for submitting a revised version of your manuscript titled "Processing load, and not stimulus evidence, determines the duration of unconscious visual feature integration". We appreciate your detailed replies to the referees. I am sorry to have to tell you that we do not feel that the editorial comments and requirements have been sufficiently addressed to justify sending this revision back to the reviewers.

In order for us to consider this manuscript further please do your best to fully address the requests below.

As mentioned in the checklists and journal guidelines, it is journal policy not to permit interpretation of anecdotal evidence; moreover, all statements and interpretations need to be based on statistical evidence or otherwise removed. The decision letter stated:
"As per journal policy, we require at least moderate evidence from Bayesian tests to support any conclusions; interpretations based on anecdotal evidence need to be substantiated by further data, or removed."

There are two issues in this regard with Experiment 3. First, the interpretation regarding the relevance of time/duration vs. number of elements is not substantiated by appropriate statistics. Instead, it's presented as if the interpretation relies solely on visual inspection:
"The left panel of Figure 5 depicts the two conditions' integration profiles when they are aligned with regard to the time at which the anti-vernier is presented. In contrast, the right panel of Figure 5 depicts the integration profiles when they are aligned with regard to the position at which the anti-vernier is presented. We see that the two curves depicted in the left panel are markedly more aligned than those depicted in the right panel. This suggests that it is the absolute time, rather than the number of lines, that determines window duration."

For the role of the SI, anecdotal evidence is provided (and appropriately described as such):"Whether, within this temporal coordinate system, the ISI has an effect on window duration merits further investigation as a two-tailed paired-sample comparison of individual subjects' slopes for the two conditions was inconclusive (revealing only anecdotal evidence with a Bayes factor of 1.303)."

The paper contains frequent statements affirming these findings and interpreting these data:
The Abstract states:"The extent of integration is further determined by absolute time instead of the number of elements presented."
In the present revision, Experiment 3 is listed under the subheading:"Integration windows depend on absolute time, rather than on the number of lines"

The Discussion includes a lengthy interpretation of Experiment 3:"Further, as revealed in our third experiment, it is the absolute time (i.e., a temporal measure quantifiable in milliseconds), not the number of lines (i.e., a measure of the number of discrete elements in the motion stream), that determines the extent of unconscious feature integration. Interestingly, this finding differs from those that have been reported in other paradigms: for instance, studies of semantic monitoring of

rapidly-presented word lists have revealed that recovery from shift costs (i.e., recovery from performance drops following a cue to shift attention) occurs as a function of the number of presented items, rather than the absolute time, following the shift cue (Alport et al., 1994; Hsieh & Alport, 1994). [...]"

We ask you to provide the appropriate statistical evidence, if necessary through additional data collection, to positively support these claims. You will find further information on our policies regarding the need to fully report appropriate statistics and limitations on interpreting evidence in this checklist: https://www.nature.com/documents/commspsychol-style-formatting-checklist-article-rr.pdf and template: https://www.nature.com/documents/commspsychol-style-formatting-guide-accept.pdf

If you are able to adequately respond to these concerns, we would be happy to look at a revised manuscript; otherwise your best course of action may be to submit elsewhere.

We hope to receive your revised version as soon as possible. If you anticipate a delay of more than three months, however, please let us know. We will be happy to consider your revision so long as nothing similar has been accepted for publication at Communications Psychology or published elsewhere.

If you are not interested in submitting a suitably revised manuscript in the future please let me know immediately so we can close your file. If you have any questions, please contact me.

Please use the link below to submit a suitably revised manuscript and updated response to referees when they are ready. As an aside, please note that in converting your manuscript file to a .pdf, the highlights tracking changes were lost. This can be avoided for example by generating your PDF in Microsoft Word with settings that protect mark-ups.

[link redacted]
** This url links to your confidential home page and associated information about manuscripts you may have submitted or be reviewing for us. If you wish to forward this email to co-authors, please delete the link to your homepage first **

In the meantime, I regret that we remain unable to publish your current manuscript in Communications Psychology. I am sorry that we cannot be more positive as things stand.

Best regards,

Marike Schiffer
on behalf of Antonia Eisenkoeck

Marike Schiffer, PhD
Chief Editor
Communications Psychology

24th May 23

Dear Mr Vogelsang,

Your manuscript titled "Processing load, and not stimulus evidence, determines the duration of unconscious visual feature integration" has now been seen by our reviewers, whose comments appear below. In light of their advice I am delighted to say that we are happy, in principle, to publish a suitably revised version in Communications Psychology under the open access CC BY license (Creative Commons Attribution v4.0 International License).

Before we can accept your manuscript for publication, we ask you to address a list of important editorial concerns and formatting requirements. These are detailed in the attached word document (the "Editorial Requests Table"). Please note that we will not be able to accept your work and send it to production unless these issues are resolved.

EDITORIAL REQUESTS:

Please review our specific editorial comments and requests regarding your manuscript in the attached "Editorial Requests Table". Please outline your response to each request in the right hand column. Please upload the completed table with your manuscript files as a Related Manuscript file.

If you have any questions or concerns about any of our requests, please do not hesitate to contact me.

SUBMISSION INFORMATION:

In order to accept your paper, we require the files listed at the end of the Editorial Requests Table; the list of required files is also available at https://www.nature.com/documents/commsj-file-checklist.pdf .

OPEN ACCESS:

Communications Psychology is a fully open access journal. Articles are made freely accessible on publication under a CC BY license (Creative Commons Attribution 4.0 International License). This license allows maximum dissemination and re-use of open access materials and is preferred by many research funding bodies.

For further information about article processing charges, open access funding, and advice and support from Nature Research, please visit https://www.nature.com/commspsychol/article-processing-charges

At acceptance, you will be provided with instructions for completing this CC BY license on behalf of all authors. This grants us the necessary permissions to publish your paper. Additionally, you will be asked to declare that all required third party permissions have been obtained, and to provide billing

information in order to pay the article-processing charge (APC).

* TRANSPARENT PEER REVIEW: Communications Psychology uses a transparent peer review system. On author request, confidential information and data can be removed from the published reviewer reports and rebuttal letters prior to publication. If you are concerned about the release of confidential data, please let us know specifically what information you would like to have removed. Please note that we cannot incorporate redactions for any other reasons.

* CODE AVAILABILITY: All Communications Psychology manuscripts must include a section titled "Code Availability" at the end of the methods section. We require that the custom analysis code supporting your conclusions is made available in a publicly accessible repository at this stage; please choose a repository that generates a digital object identifier (DOI) for the code; the link to the repository and the DOI must be included in the Code Availability statement. Publication as Supplementary Information will not suffice.

* DATA AVAILABILITY:
All Communications Psychology manuscripts must include a section titled "Data Availability" at the end of the Methods section. More information on this policy, is available in the Editorial Requests Table and at http://www.nature.com/authors/policies/data/data-availability-statements-data-citations.pdf.

Please use the following link to submit the above items:
[link redacted]
** This url links to your confidential home page and associated information about manuscripts you may have submitted or be reviewing for us. If you wish to forward this email to co-authors, please delete the link to your homepage first **

We hope to hear from you within two weeks; please let us know if you need more time.

Best regards,

Antonia Eisenkoeck

Antonia Eisenkoeck
Senior Editor
Communications Psychology

REVIEWERS' EXPERTISE:

Reviewer #1: Visual feature integration, visual psychophysics, Bayesian statistics
Reviewer #2: Visual feature integration, visual psychophysics

REVIEWERS' COMMENTS:

Reviewer #1 (Remarks to the Author):

In my previous review, I had only one major comment on the importance of the ordering of the Vernier and anti-Vernier sequence. I am glad that this comment helped the authors improve the accuracy of their illustration, and I find their answer convincing. I do not have any outstanding comment.

Reviewer #2 (Remarks to the Author):

I am satisfied with the response and revision that the authors submitted.

We are very glad to hear about the reviewers' positive evaluation of our revision and would like to thank them again for their very helpful comments.
